# Integrated Use of Measurements for the Structural Diagnosis in Historical Vaulted Buildings

**DOI:** 10.3390/s20154290

**Published:** 2020-07-31

**Authors:** Giuliana Cardani, Grigor Angjeliu

**Affiliations:** Department of Civil and Environmental Engineering, Politecnico di Milano, Piazza Leonardo da Vinci, 32, 20133 Milan, Italy; grigor.angjeliu@polimi.it

**Keywords:** structural diagnosis, data integration, structural health monitoring, digital modeling, masonry, cultural heritage, FEM

## Abstract

The process of the structural diagnosis of historical buildings is analyzed. The correlation of different data is a fundamental issue, related to the multidisciplinary nature of the study of built heritage. Quantitative data are collected by sensors, these being environmental data (temperature and humidity) or cracks (displacements). Another important source being qualitative data, derived from historic investigation, diagnostic investigations, etc. However sometimes the results may be difficult to correlate due to the different nature of the data, being quantitative and qualitative, as well as spread over the long life of the construction. In particular, the here proposed methodology suggests the use of light detection and ranging (LiDAR) scanning for the geometric and structural deformation survey, damage survey, historic evolution, monitoring of the crack pattern and environmental data. The integrated use of the collected data with digital and finite element models is investigated in two case studies. The combined use of the set of collected data is shown to be fundamental to the interpretation of the active damage mechanisms in the system, and for making appropriate decisions related to their safety. Finally, a guideline is proposed to allow for a more general use of the herein proposed structural diagnosis procedure.

## 1. Introduction

Currently, a large number of sensors, being fixed or portable, are used for the monitoring of architectural heritage and can produce a considerable quantity of data. The collected data are necessary for the comprehensive study, assessment, conservation and management of the built heritage. Among these, the structural health monitoring is of particular interest as being closely connected with the safety and the future of a building. For that reason, it is extremely important to define what measurements (forces, displacements, accelerations, temperatures and so on) have to be recorded over a certain period of time and that are considered useful to reach an understanding of the structural behavior of the historic construction [1]. Last but not least, the choice of sensors typology and their location is also important, together with the project for the installation of the data acquisition system. The structural monitoring certainly has greater potential if combined with a deep knowledge of the building, its construction history and its transformations over time, together with the necessary data resulting from the diagnostic investigation. An adequate approach for the study of historic masonry buildings must begin with the understanding of its structural logic with all its specific peculiarities and vulnerable points, together with a global survey of the damage [2].

In addition, it is always important to remember that the structural health monitoring techniques can be an efficient support to the maintenance of architectural heritage, both for professionals, before, during and after restoration works as well as for the proprietors in order to plan the necessary interventions over a period of time. Nevertheless, without a deeper knowledge of the historical construction, monitoring can provide data that are difficult to analyze, regardless of the context in which they are generated, and no strengthening intervention will possess the rational basis to ensure success [3].

In this paper, the structural diagnosis process of vaulted historical buildings is analyzed, based on an integrated approach. The correlation of different data is a fundamental issue, related to the multidisciplinary nature of the study of built heritage. The data may be provided by sensors, being environmental data (temperature and humidity) or cracks displacements, but, as above mentioned, another important source could be supplied by historic investigation, diagnostic investigations, etc. The integrated solution is required, but sometimes results are difficult to obtain due to the different nature of the collected data, being quantitative and qualitative, as well as spread over the long lifetime of the construction. In particular, here the correlation among the geometric survey and structural deformation with light detection and ranging (LiDAR) sensor, damage survey, historic evolution, non-destructive techniques, monitoring of the crack pattern and environmental data is investigated.

A demonstration is here proposed with field data of two case studies: the church of St. Bassiano in Pizzighettone (Cremona) built in the 12th century and the small church of St. Marta in Arona (Novara) built in the late 16th century. An integrated use of the collected data, in combination with digital and finite element models is studied. The combined use of the set of collected data is shown to ease the process of interpreting the active damage mechanisms in the system, and to draw important conclusions about their safety.

Finally, a flow-chart is proposed to allow for a more general use of the herein proposed diagnosis procedure. The information obtained from the proposed procedure is used either to improve the understanding of the structural response, by proposing a more refined finite element model or to take the right decisions related to the design of a long-term structural health monitoring and the conservation interventions.

## 2. Materials and Methods

A set of common practices for collecting data on historic buildings are described, with special focus on the geometric survey, damage survey, monitoring and diagnostics techniques. Elaboration of these data through digital modeling (for documentation/visualization or structural analysis), deformation analysis is further discussed in the prospective of data integration in two selected case studies.

### 2.1. Case Studies

The methods used are illustrated by two case studies: the church of St. Bassiano and the church of St. Marta.

**Figure 1:**
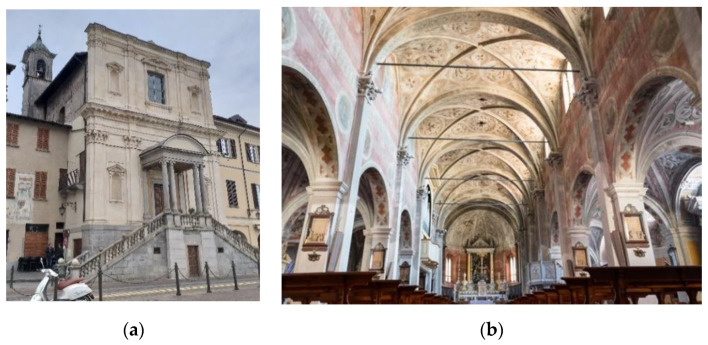
Case studies used in the present research: (**a**) St. Marta in Arona and (**b**) St. Bassiano in Pizzighettone (CR).

The small church of St. Maria di Loreto, also known as St. Marta, built in 1592, is located in Arona (Novara), on a square in the historic center overlooking the old harbor on Lake Maggiore (Figure 1a). The importance accorded to this church derives from a second construction that is enclosed within its walls: a full-scale reproduction of the Holy House of Loreto, commissioned directly by the Archbishop of Milan Federico Borromeo. The small church (24 × 10) was in such a bad condition and required restoration. The works started in 2013 on the façade, also including the restoration of the interior surfaces and the installation of a structural health monitoring system of the cracks.

The church of St. Bassiano was built in the 12th century in Lombard-Romanesque style (Figure 1b). The main dimensions in plan are 25 m × 40 m. The span of the nave is 6.8 m, which is twice the span of the aisle. The church is located in the center of Pizzighettone, a small city in Northern Italy along the Adda river. The church of St. Bassiano was closed for celebrations and access for visitors in 2012 due to significant damage observed in the structure.

### 2.2. Geometrical Survey and Modelling

#### 2.2.1. Survey

The role of the geometric survey discussed in this section is apt to obtain measurements for digital modeling and the structural diagnosis purpose.

In the case of simple structural elements such as walls or piers, the survey of the observed geometry can be carried out with simple tools. While in the case of curved elements (vaults) or remote parts, sophisticated survey technology such as photogrammetry or laser scanners have a clear advantage over classical methods [4]. LiDAR (light detection and ranging) measurements introduced a new perspective for a rapid fast survey of historic buildings. This remote sensing method uses light in the form of laser pulses to measure point coordinates with respect to the sensor origin. It is particularly useful when dealing with complex structural elements like masonry vaults, since the structural response is closely connected with the possibility of considering the real vault shape for a structural analysis. A major drawback is that only the visible part of the structure can be captured as a point-cloud. Within this context, direct methods of inspection are still very important, since they allow for a direct description of the internal section of the structural elements [5].

**Figure 2:**
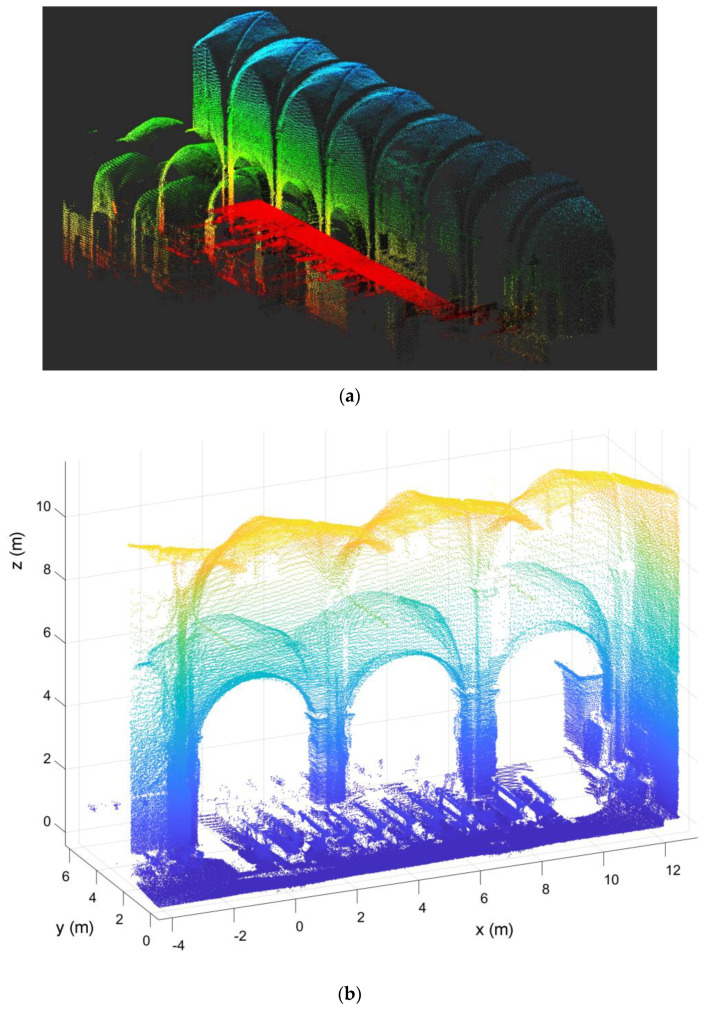
Church of St. Bassiano: point cloud light detection and ranging (LiDAR) survey: (**a**) the complete survey and (**b**) detailed view of the first three bays, toward the façade.

**Figure 3:**
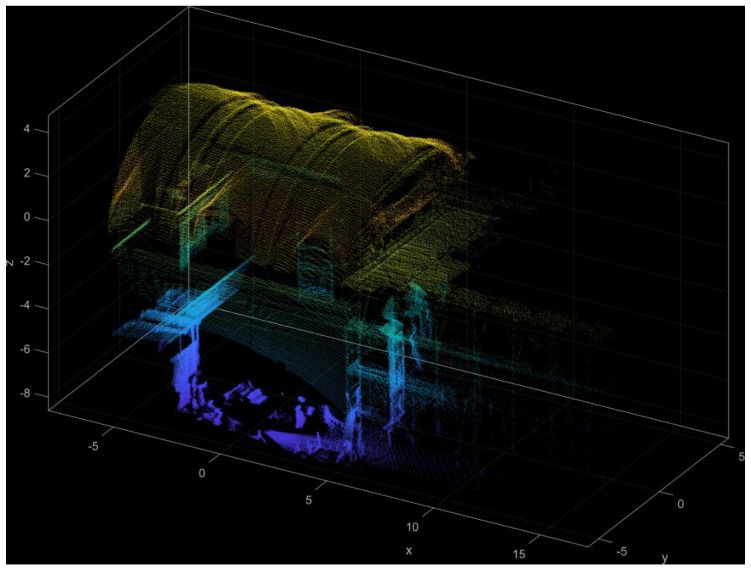
Church of St. Marta: point cloud LiDAR survey (only the first 3 bays were surveyed).

Examples of geometric survey using LiDAR sensor in the churches of St. Bassiano and St. Marta are shown in Figure 2 and Figure 3. Both surveys were carried out using a high-resolution LiDAR sensor (OS1-64) with a maximum range of 80–120 m depending on surface reflectivity. During the survey with the LiDAR sensor it is possible to view the scan in real time, which allows one to minimize problems of missing parts due to obstacles in the post-processing phase. This feature was very helpful in both cases since the registration was carried out during restoration works, with many parts of the building encumbered by scaffolding.

The configuration during the acquisition phase was set out to a horizontal resolution of 2048 points (equivalent to 0.18° angular resolution) and a vertical resolution of 64 point (equivalent to 0.35° angular resolution) at a rotation rate of 10 Hz. The output of the surveying process is several scan frames in the unit of time, e.g., 10 frames per second in the present case. A part of these scans is selected and used later to create the final point cloud. Usually, the more frames that are used, the better the quality of the survey. However, it is not necessary to use all the scan frames since the alignment process becomes very time consuming and most of the scans contain similar geometric data, so a part can be omitted. After a set of scan frames suitable for the reconstruction has been selected, a two-step alignment process is necessary. The initial alignment was based on the data provided by the inertial measurement unit. A second alignment step was carried out by using the iterative closest point algorithm (ICP) [6]. In general, the alignment error in the final point cloud varies between 0.015 and 0.025 m.

In the church of St. Bassiano, a continuous path along the central nave was considered, from which only 12 scans out of 150 were extracted and used to create the final point cloud. The final point cloud includes 1 million points and an alignment error of 0.025 m (Figure 2a). A detailed view of the first three bays is shown in Figure 2b.

In the church of St. Marta, the objective was to analyze the masonry vault, so only 2 scans were extracted and used to create the final point cloud (Figure 3). The final point cloud included approximately 60,000 points and an alignment error of 0.015 m.

The final aim of the registered point cloud was to extract geometrical measurements for the modeling process. While the results were straight forward for simple elements such as walls or pillars, the measurement became very complex for curved elements like arches or vaults. In which case, parts of the point cloud need to be further elaborated in order to extract geometric quantities as radii or a centre of arches. The identification of the geometric shapes in historic buildings was discussed previously in detail in [7].

The procedure is based on a mathematical fitting of the equation of a circular or an elliptic arch in a subset of the registered point cloud [8]. The data can be used for manual geometric modeling of the vault or for understanding the structural deformations.

**Figure 4:**
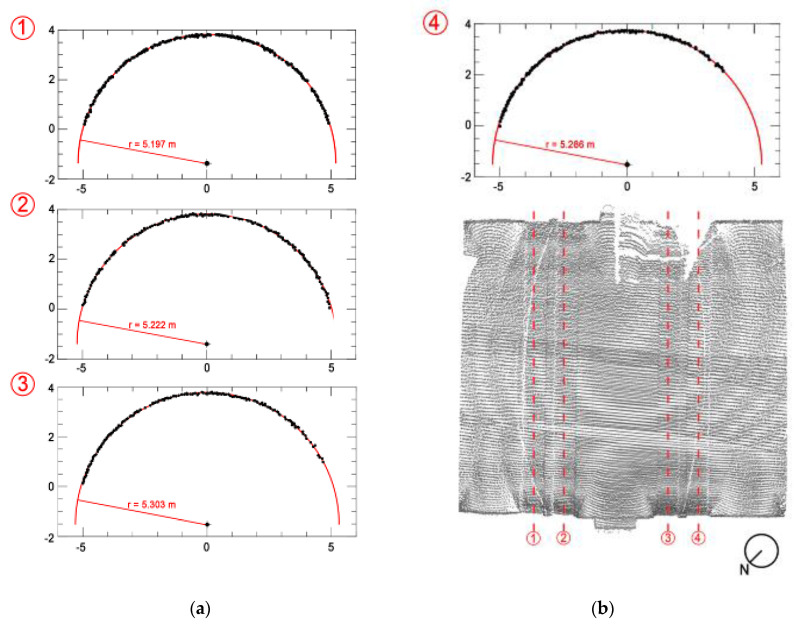
Church of St. Marta: identification of geometric shape of the twin arches by least square minimization procedure: (**a**) four transversal sections of the arches extracted from the point cloud and (**b**) localization in a plan view of the extractions.

An example from the vaults of the church of St. Marta is shown in Figure 4. Four sections were extracted from the point cloud, in order to understand the geometric shape of the twin arches. It is noted that there is a difference of about 3 cm between the first and second arch, 8 cm between the second and third arch and 2 cm between the third and fourth arch (Figure 4). More advanced procedures involving classification algorithms are considered by other researchers to detect changes and anomalies in buildings [9,10,11,12].

#### 2.2.2. Geometric and Structural Modeling

**Figure 5:**
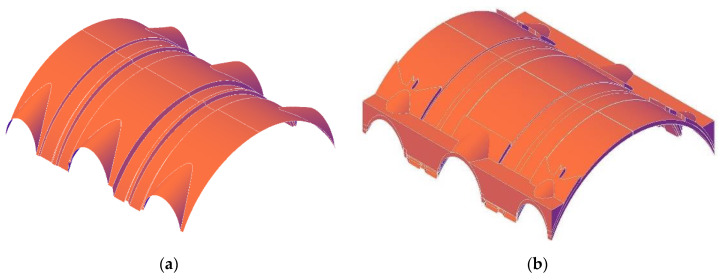
Digital geometric model of St. Marta church vaults for visualization and documentation: (**a**) intrados survey and (**b**) volumetric representation, observed from the extrados.

The registered point cloud cannot be used in a straightforward way to obtain a geometrical model. In general, the geometric model can be developed over the point cloud in three ways: (a) manual modeling [13,14], (b) parametric modeling based on user defined inputs [7,15,16] and (c) automatic reconstruction methods, mostly based on point cloud segmentation and surface fitting methods over a set of point clouds [5,17,18,19,20]. Manual modeling allows the creation of very detailed models (including the thickness of the sectioned structural elements, which cannot be surveyed with LiDAR scanning), but this is very time consuming. Whereas, automatic reconstruction methods are very fast, provide high geometric accuracy on the exterior surface, while remaining of poor quality for the reconstruction of the elements thickness. In Figure 5a there is shown a simple reconstruction of the barrel vault, based on the surface record taken from the point cloud, using an average thickness of 20 cm. However, direct inspection on site has shown that it does not have a constant thickness. These details are modeled manually enriching the geometrical model with details from the inspection (Figure 5b). In the extrados a groove is present above the twin arches, which is 170 cm wide. It creates space for the timber trusses over the barrel vault that would otherwise lie directly on the vault (Figure 5).

**Figure 6:**
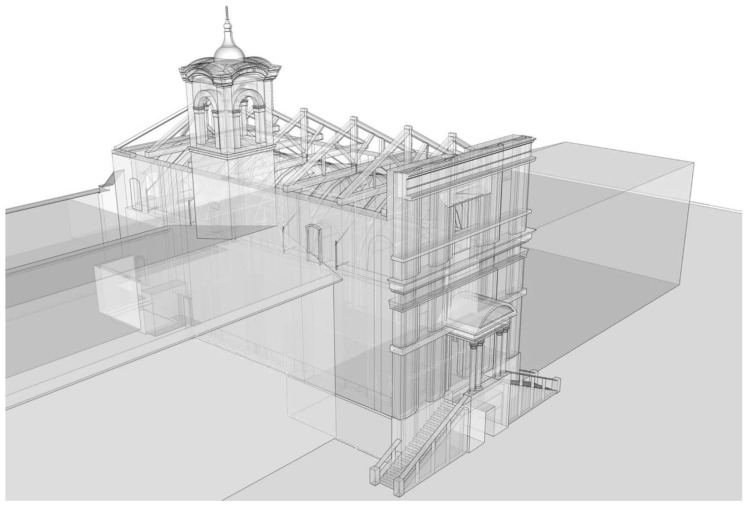
Global digital geometric model of St. Marta church for visualization and documentation.

Finally, a global digital model for visualization and documentation purpose of St. Marta church is shown in Figure 6. The model was created using SketchUp software and includes most of the architectural details recorded by LiDAR scanning as well as those obtained during the visual inspection process. On the other hand, a numerical model cannot have the same geometric accuracy as a visualization model, as it should represent the structural behavior with suitable accuracy.

In particular, to generate the geometry of detailed structural models is more complex compared to other kinds of digital models as more constraints apply during their development phase, e.g., modeling of internal divisions related to material properties, geometric continuity (3D solid parts should be in full contact between each other, which is usually neglected in a purely geometrical model), geometric intersections must be avoided, strict meshing requirements as well as being computationally expensive during analysis [4,21].

**Figure 7:**
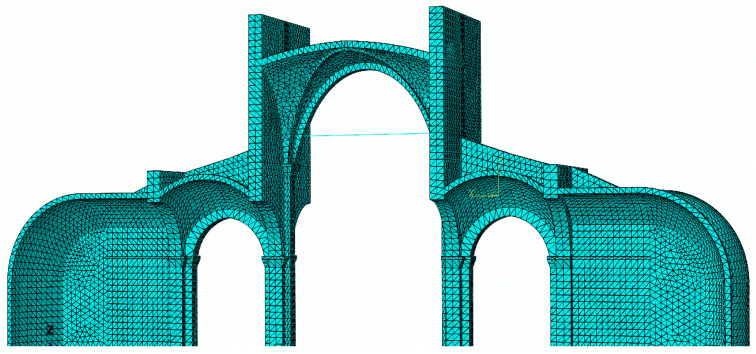
St. Bassiano church: numerical model for structural analysis, representing a transversal slice of one bay with the nave, two aisles and two external chapels.

In Figure 7 is shown the representative finite element (FE) model of one typical bay of St. Bassiano church. The geometry is based on LiDAR scanning of the church interior (see Section 2.2.1). The FE model includes approximately 300,000 nodes, and a discretization with 122,000 linear tetrahedral elements. The model considers a parametric geometric modeling procedure, for the vault in the central nave, which is the most complex parts of the system [7,22]. The rest of the structural members (the piers, the buttresses, the walls and iron tie rods) are modeled manually in Abaqus CAE release 6.14 [23]. The masonry material is modeled with a plastic–damage model proposed by Lubliner et al. [24] and by Lee and Fenves [25], implemented in the software Abaqus. We assumed the masonry strength in compression fc, 4 MPa, strength in tension ft, 0.2 MPa, and the modulus of Elasticity E, 2000 MPa. Lower values were used for the mechanical properties of the rubble fill in masonry vaults, fc = 2 MPa, ft = 0.15 MPa and E = 800 MPa. This is a macroscopic approach where cracking is considered in an average sense.

### 2.3. Deformation Analysis

**Figure 8:**
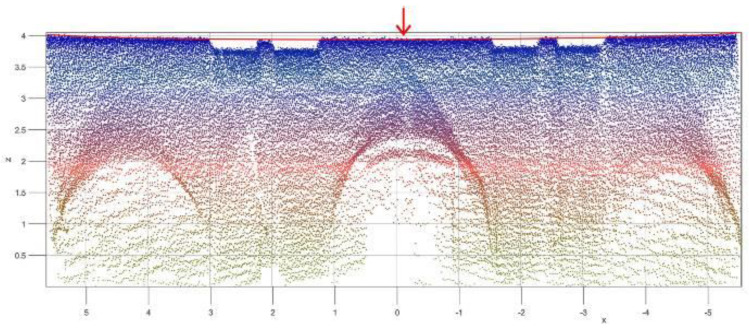
Side view of the point cloud with deformation indication.

The differences between the identification of the real irregular geometric shapes and the supposed regular geometry can be attributed to a deformation. As in the case of St. Marta church, the small differences surveyed in the three vaults, which must be further investigated, can be probably related to construction irregularities, due to the subsequent addition of the vault, or to structural deformations. The average thickness of the barrel vault is 20 cm. A longitudinal section of the point cloud allows one to see a 3–4 cm deflection extended between the 1st and 2nd bay (Figure 8).

### 2.4. Construction History

**Figure 9:**
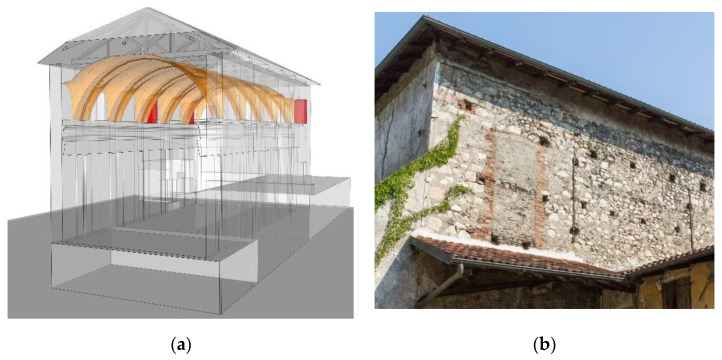
(**a**) Drawing of the church of St. Marta after the masonry vault has been added and with the windows paneled highlighted in red and (**b**) one of the paneled windows.

The irregularities can so be compared with data from archives, which confirmed in the previous case that in St. Marta church the barrel vault was inserted at a later date, after an interruption of works and after the roofing and external windows had already been completed (Figure 9).

**Figure 10:**
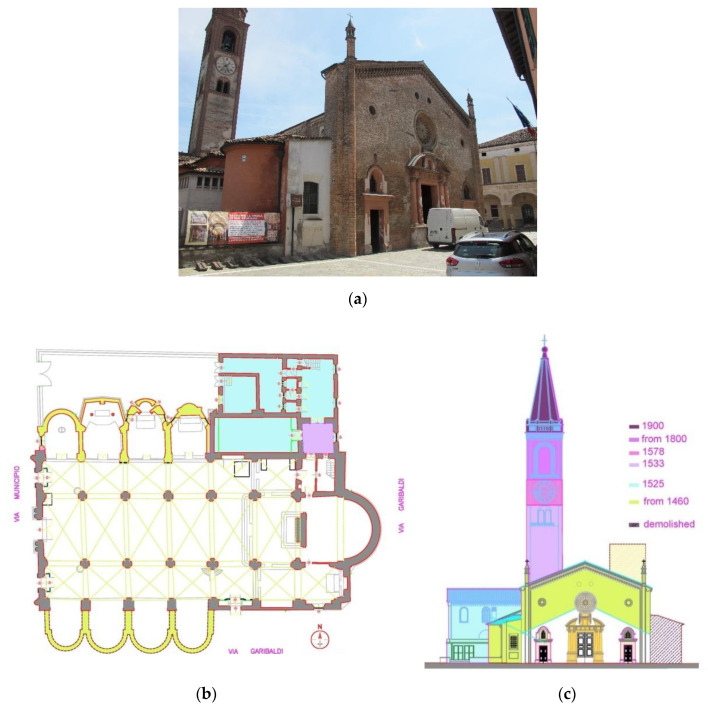
Historical evolution of the St. Bassiano church: (**a**) façade photo (**b**) historical evolution plan and (**c**) historical evolution elevation.

As an example of a very complicated construction history, we would like to mention the St. Bassiano church in Pizzighettone (Cremona). The structural evolution of the church of St. Bassiano is closely connected with historic events between the 15th and 19th centuries, when it was the object of many interventions [3] (see Figure 10).

In the 15th century, Pizzighettone became part of the Milanese domain first under the Visconti and then the Sforza family, who built the beautiful masonry town walls [26] and radically transformed the architectonical aspect of the church: the façade was elevated, and a rose window was inserted by cutting a pre-existing window. Two rows of chapels were added on both external sides of the aisles (to the north and the south). A new roof covered the nave northwards and the chapels with only one single roof pitch.

During the time of the late Renaissance, between 1525 and 1580, the sacristy was added north-west of the church; the bell tower was moved from the south to the north side of the apse and the first part of the present tower was built; in 1578 the first elevation of the belfry took place in order to add a clock.

In the 19th century the south-facing chapels were demolished, probably due to the need to widen the adjacent roadway. At the same time, iron tie rods were added to the masonry vaults. In 1820 the belfry on the north side was further elevated and in 1835 the interior of the church was fully decorated in the present visible style.

In the early 20th century the final elevation of the octagonally shaped belfry was added. In 1963 a restoration work carried out by the architect A. Edallo, changed the roof, in part with modern reinforced concrete beams, and the long single pitch northward was demolished. Two chapels on the north side were rebuilt in modern style.

In this context it is important to note that the absence of material homogeneity and uniformity of design makes the construction more vulnerable from the structural point of view, compared to that of a building that has never undergone structural modifications over the years.

### 2.5. Damage Survey

The crack pattern in St. Bassiano church survey highlighted the seriousness of the damages both to the vertical structures, weakened by the continuous alterations over the centuries, and in all the vaults. The belfry and the sacristy are seriously damaged too.

The past interventions on the roof, with its thrust, have put a burden on the nave vaults web and subsequently on the aisles, causing many structural cracks and detachment from the arches of the central nave. The different elevations of the northern belfry, caused a relevant out of plumb of about 12 cm towards the north-east side, with resultant crushing on one side and dragging on the other side, with consequences on the sacristy. Many cracks due to masonry discontinuities are present in the chapels and a soil settlement effect is recognizable on an internal pillar of the main nave in south-east direction. The western façade showed an out of plumb of the gable outwards.

**Figure 11:**
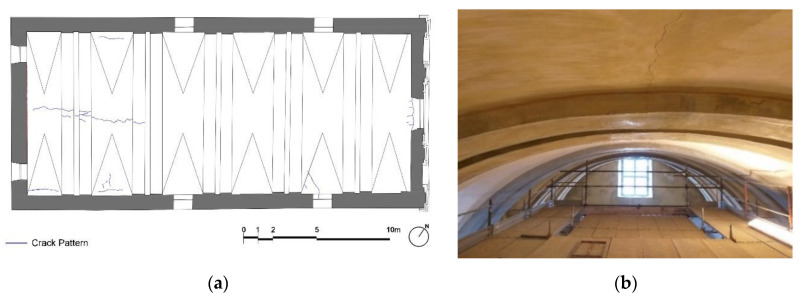
St. Marta church: (**a**) plan of the barrel vault and (**b**) irregular masonry barrel vault observed from the scaffold at a height of about 9 m. A longitudinal crack is visible at the center.

Inside the church of St. Marta, a non-passing through crack is visible on the ceiling of the vault that runs along the key of the vault (Figure 11). Cracks are also present at the lower level of the church (westward) along the longitudinal walls, highlighting the excessive vault thrust, not balanced by the iron tie-rods, which appear to be poorly tensioned.

### 2.6. Monitoring

As already mentioned above, the structural health monitoring techniques can offer an efficient support to the maintenance of architectural heritage, here a peculiar structural health monitoring system was installed in St. Bassiano church.

St. Bassiano church was closed down for celebrations and access for visitors in 2012, as the damage observed in the structure had seriously increased after the earthquake that hit the Emilia Romagna region in 2012. Due to the seriousness of the surveyed damages, a complete diagnostic investigations campaign was firstly carried out on the church structures, together with a short period monitoring of a few selected cracks on the apse [3]. Then the structural intervention was planned and it was not only economically problematic but required the closure of the town’s main church for many years. The decision taken to subdivide the intervention in different phases became a necessity: a first phase was considered urgent, allowing the temporary reopening of the church at the end of this step; a second and a third phase was postponed to raise the necessary funds and to bring the interventions to its conclusion.

After the first intervention phase and while waiting for the two subsequent phases, the crack pattern was constantly controlled by a remote structural health monitoring [3], in order to: (a) control the remaining unrepaired parts during the time that occurred for raising funds; (b) evaluate the efficiency of the first phase of intervention over a period of time and its effect on the remaining damaged portions and (c) reopening the church to the faithful in safety conditions without leaving props or other obstacles, giving the important signal to close the building as soon as movements are measured over a certain risk level threshold.

**Figure 12:**
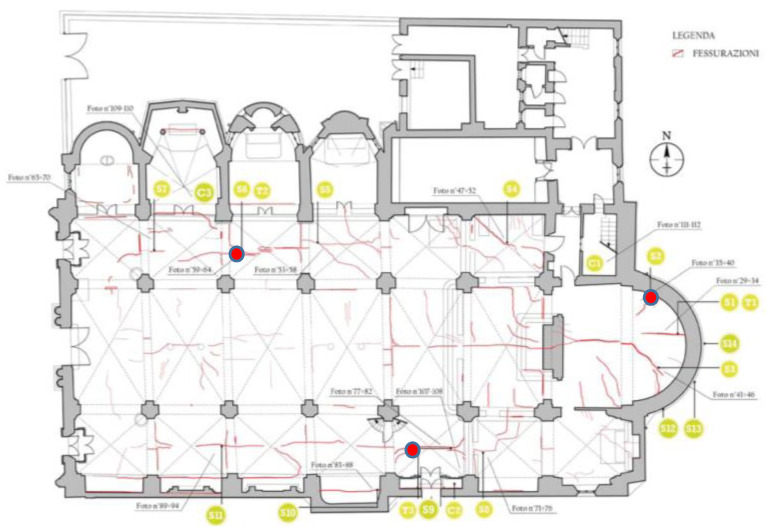
Plan of the St. Bassiano church with representation of the crack pattern and sensor location. The three red points represent the monitored cracks reported in the next Figure 13 and Figure 14.

Some cracks have been selected and located in the unrepaired areas, as the groin vaults of the aisles. Some monitored cracks are located also in the apse both internally to verify the efficiency of the realized intervention and externally to verify the effect of the continuous vibrations caused by traffic (Figure 12).

**Figure 13:**
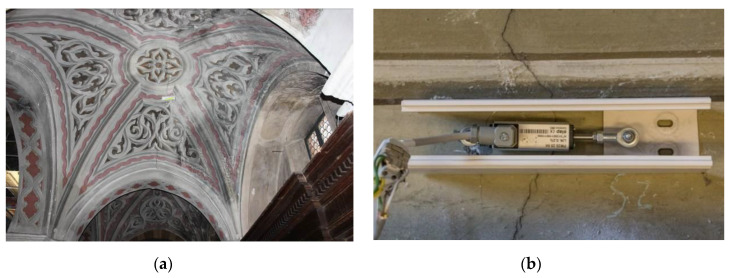
St. Bassiano: (**a**) monitoring of cracks in a masonry vault by means of (**b**) LVDT displacement transducers.

Cracks are monitored with linear displacement transducers (Figure 13), sending signals to a data logger, which converts signals and sends them constantly through the phone line. Measurements were recorded every minute and averaged to half an hour, since 2014. Usually, a period of at least 18 months is long enough to evaluate the active movements, their velocity, the effects of additional damage causes still un arrested, such as the soil settlements, and the daily thermo-hygrometric vibrations.

**Figure 14:**
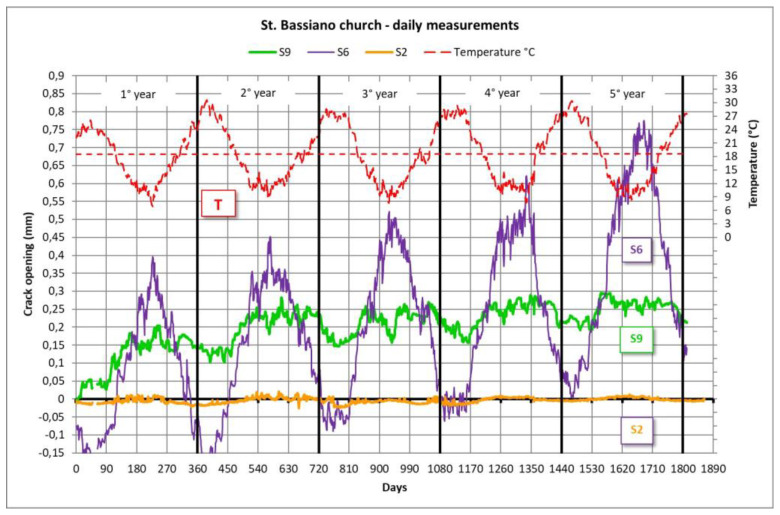
St. Bassiano church: plot of the 5-year crack monitoring, after the strengthening intervention on the nave vaults, apse and roof. Only some significant sensors are plotted.

Although present large oscillations on the external side, the cracks monitored inside the apse are stable and confirm the strengthening intervention efficiency (S2 sensor). The monitored cracks on the aisles, due to the first phase intervention on the nave, decreased their opening, except for a couple of cracks in the south aisle (here only S9 sensor is reported) and one in the north aisle (S6 sensor; Figure 14). The trend of S6 against the temperature shows that this crack is passing through the masonry wing of the vault: as the temperature increases, the masonry dilates and the two edges of the crack come closer together; as the temperature decreases, the masonry shrinks and the crack opens. Unfortunately, this oscillating movement is not stable and, year after year, it shows a clear trend for the crack to open over time. On the contrary, S9 was very concerned for the first two years of monitoring and then seems to stabilize in the following years.

## 3. Results and Applications

In this section some results are presented, showing the possibility of reading the collected data and measurements in an integrated way. Three categories were considered: (a) visual integration of data, (b) integration of historical information within numerical models and (c) integration of sensor measurements with simulation models.

### 3.1. Visual Data Integration

Visualization of collected data, in a graphic form over geometric models, is the simplest form of representation. While for an experienced professional it could be easier to combine information, presenting key information in a readable way, in the case of an inexperienced user it is a significant benefit.

**Figure 15:**
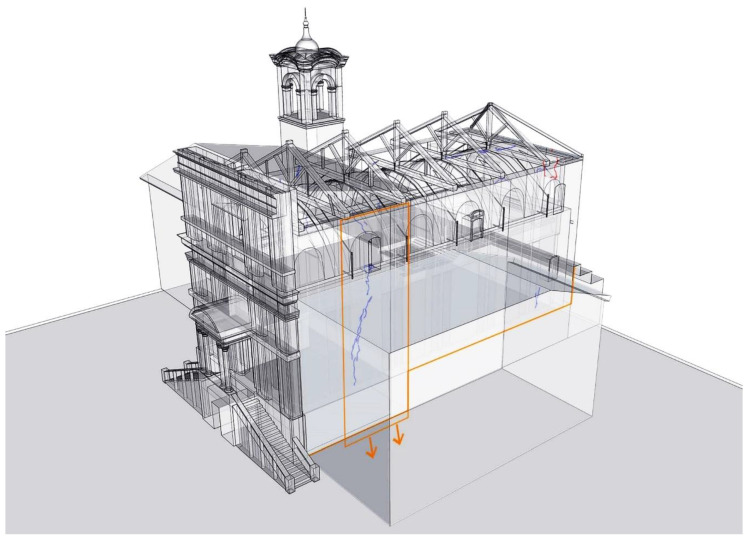
Visual representation of the relation between the observed damage and soil-settlements.

In Figure 15, a visual representation of the crack pattern over the geometric model of the church of St. Marta is presented. The visualization helps to understand clearly the relation between the observed damage and soil-settlements, below the Holy House of Loreto. The presented information of this is organized over various layers and can be visually combined. Another combination could be to visualize the information combined with the building evolution over a time.

### 3.2. Integration of History of Construction within Numerical Models

In this section, the integration of the results of the historical analysis was explained within a simulation model. The importance of considering the structural evolution in time, relates to the fact that it provides essential information on past collapses, restorations or the presence of certain structural elements, which cannot be explained differently (see Section 2.4). For instance, in the case of the church of St. Bassiano presented in this paper, the presence of the iron tie in the nave can be explained by the removal of the southern chapels in the 19th century [3].

**Figure 16:**
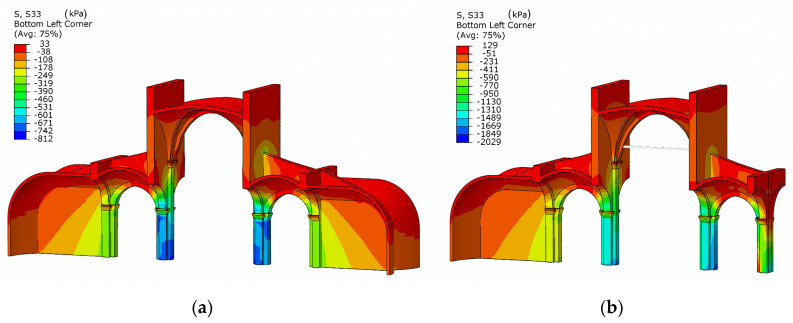
St. Bassiano church: example of considering the structural evolution through sequential analysis in a numerical FE model. (**a**) Vertical stresses in the configuration with both chapels and (**b**) vertical stresses in the configuration after the removal of the southern aisle.

**Figure 17:**
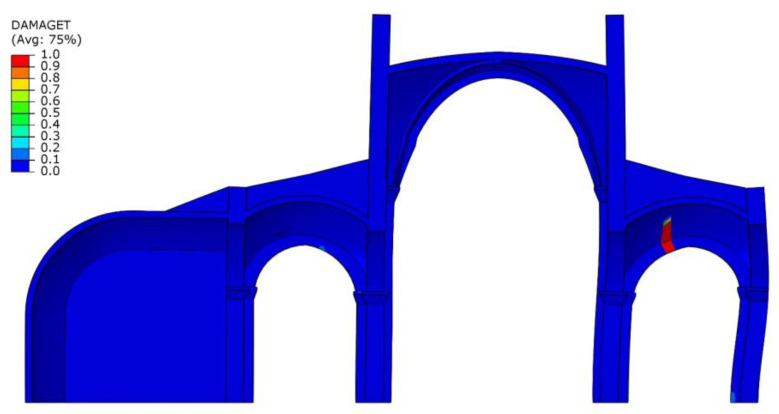
Damage in the southern aisle due to the removal of the chapel in the current configuration.

In order to integrate this information within the numerical model, the structural analysis considers a sequential analysis focusing on the interventions in the 19th century, where the southern chapels were removed, and the central nave was reinforced with iron ties to contain its horizontal thrust (Figure 16). Additionally, previous research has shown that the consideration of the construction stages within the analysis is important for an improved prediction of the axial force in iron ties [15]. The results show that after the removal of the southern chapels the stress level increased considerably in the nave piers and the vertical stress distribution became asymmetric (Figure 16). When analyzing the actual damage for self-weight conditions, it cannot be predicted in the original configuration with both chapels. In difference to the original configuration with both chapels, in the current configuration (considering the structural evolution in time, with asymmetric chapels) new damage is predicted only in the southern aisle (Figure 17).

### 3.3. Integrated Use of Virtul and Structural Models with Sensors

The combined use of simulation model and a monitoring system is explained in this section. While most of the monitored cracks on the aisles, due to the first phase of the strengthening intervention on the nave, decreased their opening excepting a couple of cracks in the south aisle (S9, S10) and one in the north aisle (S6). In particular, the S9 measurement base, located near the south-eastward first pillar, has shown a constant opening increase over the time mainly due to a differential soil settlement, without showing a strong effect of seasonal variations. S6 on the north aisle, despite the great seasonal variations, continues to open up (Figure 14).

Using the simulation model, several scenarios were developed based on the settlement of each of the supports, producing opening and closing of certain cracks. Among them the scenario with settlement of north support (the buttressing proved by the chapels) matches the results of the monitoring system.

**Figure 18:**
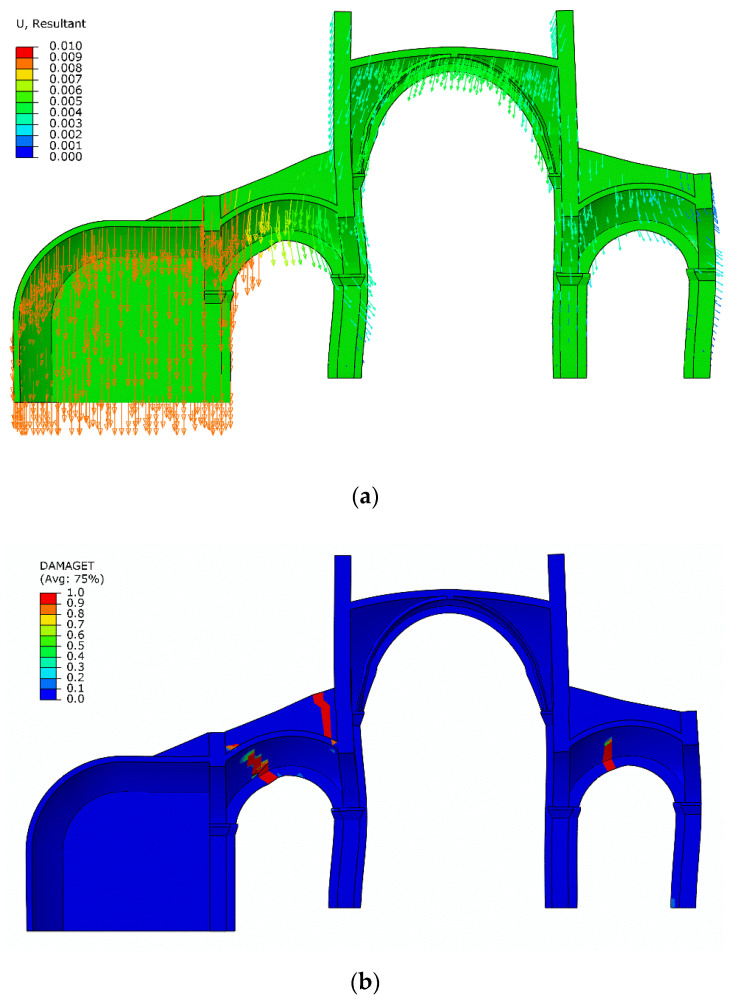
Simulation of support settlement: (**a**) displacements and (**b**) damage in tension.

In this scenario, the crack monitored on the northern aisle continues to open, while the crack on the southern side is nearly unaffected, maintaining the same level of damage (Figure 18a). This is clearer when comparing damage prediction under tension before and after settlements (Figure 17 and Figure 18b). It is obvious that only the damage on the northern side monitored by sensor S6 continued to evolve while the damage under tension on the southern side remained more or less the same (compare Figure 17 and Figure 18b).

## 4. Discussion and Conclusions

Of late, the investigation process in a historic building has clearly become multidisciplinary. When considering the collected data in historical buildings, it is important to use them as efficiently as possible. In this paper it is discussed the possibility of making a combined use of multidisciplinary data for the structural diagnosis of historical vaulted constructions.

Typical collected data analyzed in most projects are a laser scan geometric survey, inspection, crack pattern survey and deformation analysis of certain structural elements, while in some advanced cases numerical models and a monitoring system.

The two inserted cases studies were shown to be important in illustrating the proposed strategies of integration. The aim is to discuss standard and advanced techniques of data integration and to improve the efficiency of its use in the decision-making process.

Among the standard strategies is the visual integration of the data. This technique, is quite diffused currently in the state of the art, and is based on the division of various information in various layers and the combined view based on the necessary requirements. The clear interest here is an improved understanding, in particular for non-experienced analysts.

The use of finite element models is discussed in the prospective of advanced integration. The finite element model presented in this paper was developed in Abaqus software. It includes a nonlinear modeling of the masonry material with a plastic damage model, which is essential in simulating cracking. The application in the case of St. Bassiano church illustrates how the structural evolution in time (understood through the historical analysis) can be integrated in a FE model though a sequential analysis. Important phases in the construction such as the removal of the southern chapels and the insertion of the iron ties are considered in the numerical simulations. This produces a more realistic state of the stress in the numerical model compared to the instantaneous application of the loading (as considered traditionally). This is helpful in understanding damage visible in the southern aisle that could not be simulated numerically otherwise, and the tension values of the iron tie-rods obtained during the diagnostic phase.

The third integration possibility is to combine the simulation model with the monitoring system. Although it was clear from the start that the damage was connected to soil settlements, it was possible to understand specifically which support settlement was active in the monitored period. The integration in this case was considered by producing several settlements scenarios with the FE model and matching the one producing similar damage with the one reported by the displacement sensors.

In particular the two last applications, which consider very detailed structural models have been introduced recently in the literature with the term Simulation Digital Twin [27].

**Figure 19:**
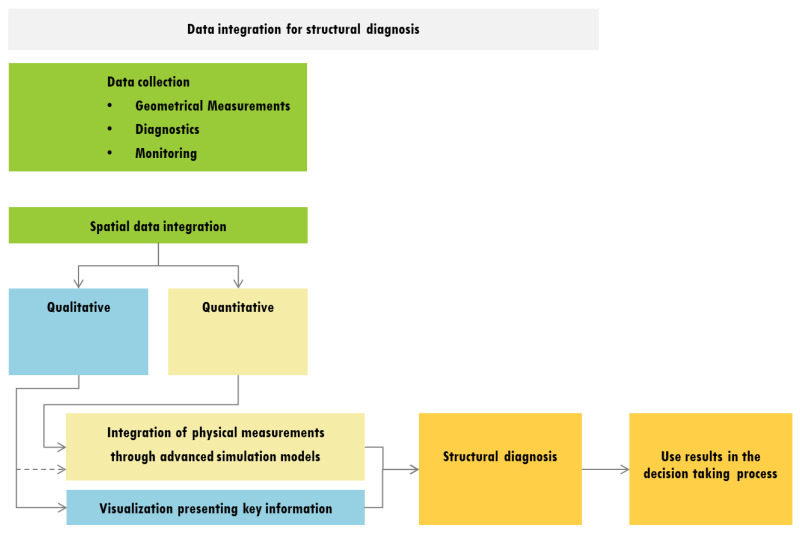
Proposed methodological approach for data integration for structural diagnosis.

A summary of the considered methodological approach for data integration and its use for the structural diagnosis is shown in the form of a flow chart in Figure 19. The created set of data is of a multidisciplinary nature. After the creation of this database it is essential to divide them in two categories: qualitative and quantitative. As described in Section 3, each of the two categories can be integrated in a different manner. Qualitative data as historical analysis, crack patter, material degradation, etc., can mostly be integrated in a geometrical model aimed for visualization purpose. Quantitative data, as geometry, deformations of structural elements, mechanical parameters or other can be integrated by means of a finite element model. In this aspect another contribution of this paper was to better use the qualitative data as the history of construction by integrating it in the finite element model.

Finally, most of the collected information including geometry, damage, monitoring and history of construction involves largely manual processing steps. These are very time-consuming processes, which also include a good deal of interaction between project members. This is a major obstacle in the current integration process of various information. Further work could focus particularly on the automation of the data collection process and its structured organization. Recent technologies such as laser scanning, building information modeling or Digital Twin could play a major role in this process.
